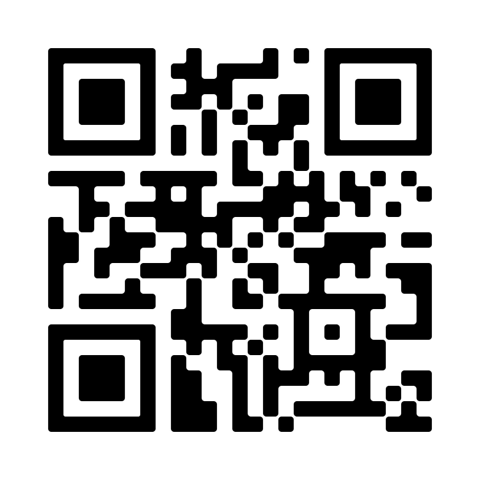# Incorporating 3D reconstruction in preoperative surgical planning of Multiple Myomectomy

**DOI:** 10.52054/FVVO.14.1.009

**Published:** 2022-04-03

**Authors:** G Armano, S Barbuto, S Wagner, J Carugno, G Bifulco, A Di Spiezio Sardo

**Affiliations:** Department of Medicine and Surgery, University of Parma, Parma, 43125, Italy; Medics SRL, Moncalieri, Turin,10024 Italy; Obstetrics, Gynecology and Reproductive Sciences Department. Minimally Invasive Gynecology Division. University of Miami, 33136, FL USA; Obstetrics, Gynecology and Reproductive Sciences Department. Minimally Invasive Gynecology Division. University of Miami, 33136, FL USA; Department of Neuroscience, Reproductive Sciences and Dentistry, School of Medicine, University of Naples “Federico II” Naples, 80131, Italy; Department of Public Health, School of Medicine, University of Naples “Federico II” Naples, 80131, Italy

**Keywords:** Myomectomy, myoma, fibroid, 3D reconstruction, hyper accuracy

## Abstract

**Background:**

Medical 3D imaging is a promising emerging technology that allows recreating the details of human anatomy. The use of this innovative technology has resulted in improved surgical efficiency and better clinical outcomes. However, its incorporation in gynaecologic surgery has not been widely adopted.

**Objectives:**

To demonstrate the use of Hyper Accuracy 3D reconstruction in a patient with infertility who underwent multiple myomectomy.

**Materials and Methods:**

A stepwise approach describing the incorporation of Hyper Accuracy 3D imaging technology into the preoperative surgical planning and intraoperative guidance of a patient with multiple myomas undergoing multiple myomectomy.

**Main outcome measures:**

Preoperative evaluation of a patient with multiple myoma and infertility who presented to our department seeking surgical management. Hyper Accuracy 3D image was obtained, and a 3D digital image reconstruction of the uterus delineating the exact number, volume, and location of the fibroids was created. The 3D digital image was available during the surgical procedure which helped to plan the surgical steps allowing a systematic surgical approach resulting in an effective surgery with minimal blood loss.

**Results:**

The benefits of intraoperative guidance using Hyper Accuracy 3D in a patient with multiple myomas and infertility are demonstrated.

**Conclusions:**

The adoption of this promising imaging technology into gynaecologic surgery is feasible and should be further investigated. Additional studies evaluating the clinical impact of using Hyper Accuracy 3D imaging in the preoperative planning of patients with gynaecologic surgical pathology are needed.

## Learning objective

The video article aims to demonstrate the use of Hyper Accuracy 3D ® (HA3D) technology in an infertile patient who underwent multiple myomectomy. In complex surgical cases, identifying all myomas during surgery can be challenging. The use of HA3D technology helps the surgeon during the preoperative planning of complex cases. A Step- by-step description of the use of HA3D technology in a complex case of multiple myomectomy in a patient with infertility is presented.

## Introduction

Uterine myoma is the most common benign gynaecologic tumor in reproductive-aged women (ACOG Practice Bulletin, Number 228,2021; Giuliani et al., 2020).

The management of uterine fibroids is dictated by their number, size, location, patient’s age, and desire to preserve fertility (ACOG Practice Bulletin, Number 228,2021; Donnez and Dolmans, 2016). In women with multiple myomas who wish to preserve their fertility, myomectomy is a useful surgical option (ACOG Practice Bulletin, Number 228,2021; Donnez and Dolmans, 2016; Giuliani et al., 2020). Unfortunately, imaging diagnostic techniques are not always available to the surgeon during the surgical procedure, and some potentially resectable fibroids may not be detected during surgery (Levens et al., 2009). The HA3D technology represents the latest technological evolution of traditional diagnostic tools to process two-dimensional radiological images as Magnetic Resonance Imaging (MRI) and Computed Tomography (CT) into three-dimensional models.

Lately, different authors reported the benefits of incorporating HA3D imaging in preoperative surgical planning and intraoperative guidance (Pan et al., 2021; Sallent et al., 2017). The interactive three- dimensional representation is a promising emerging technology that provides real time anatomical reconstruction allowing to tailor the best surgical strategy. The use of this innovative technology has resulted in improved surgical efficiency and better clinical outcomes (Chae et al., 2021). The use of HA3D has not been widely adopted in gynaecologic surgery (Martelli et al., 2016).

## Patients and methods

We present a complex case of a 35-year-old nulliparous infertile woman with multiple uterine myomas. The patient underwent laparotomy with multiple myomectomy assisted by preoperative HA3D-reconstruction at the Department of Public Health, School of Medicine, University of Naples “Federico II” Naples, Italy.

The HA3D workflow to obtain 3D reconstruction required different steps. At first, a two-dimensional image acquisition (MRI with a 3 mm slice thickness) was obtained. Then, a DICOM format image was processed through a radiologic acquisition protocol optimised for specific pathology. Following, a qualified bioengineer carried out the segmentation through a CE marked medical-grade software (Mimics Medical 21.0, materialize, Leuven, Belgium). Finally, the segmented voxels were converted into surface mesh and a virtual 3D model was obtained.

In our case, the HA3D technology allowed the identification of 25 fibroids. A mapping of the myomas was carried out first. Then, the size and location of each myoma in relation to the uterine cavity were determined to provide additional information to the surgeon.

A real-time 3D reconstruction was obtained to guide the surgeon during the surgery. Outside the operating field, an assistant manipulated the 3D reconstruction file synchronously with the surgeons. Details of the surgical procedure were documented.

## Results

The HA3D technology allowed to preoperatively identify 25 myomas. Nineteen myomas were removed during surgery with only 50 mL of intraoperative blood loss. Six myomas were not planned to be removed due to their small size (<10 mm). During surgery, the intramural myoma number 9 was non palpable. Guided by the HA3D technology, the surgeon was able to locate and easily remove the non palpable myoma without injuring the surrounding healthy myometrium. The total operative time was 90 min.

## Discussion

The present video article describes the feasibility of using preoperative hyper accuracy 3D imaging technology to guide the surgeon during the intervention. Despite the large number of myomas that were removed, the procedure was performed in a relatively short operative time (90 minutes) with minimal blood loss (50 mL).

To date, a growing number of studies reported the benefits of HA3D in preoperative surgical planning and intraoperative guidance in non-gynaecological procedures (Tsaturyan et al.,2021; Porpiglia et al., 2018; Porpiglia et al., 2019). However, only a few studies have described the 3D imaging technique feasibility in the gynaecological field (Lee et al., 2018; Aluwee et al., 2015). To the best of our knowledge, this is the first video-article describing the use of HA3D imaging technology in gynaecologic surgery.

In daily clinical practice, traditional diagnostic techniques have the major limitation of not being available intraoperatively (Levens et al., 2009). Several authors use intraoperative transvaginal ultrasound to overcome this limitation. However, the transvaginal ultrasound probe requires the use of sterile probe-covers and is time-consuming, expensive, and not always available (Shigeta et al., 2020). Moreover, ultrasound has limited effectiveness for detecting myomas smaller than 5 mm in diameter (Shigeta et al., 2020).

Conversely, HA3D technology ensures the identification of myometrial lesions as small as 5 mm diameter. Furthermore, the intraoperative image re-processing is performed by an assistant outside of the surgical field maintaining the sterility of the surgical field throughout the procedure.

It is our opinion that the best advantage of incorporating this innovative technology would be in cases performed with a minimally invasive endoscopic approach, especially in cases performed with robotic technology in which the lack of haptic sensation increases potential difficulty in the localising the fibroids. However, as demonstrated in our case, the use of HA3D technology is also useful when dealing with multiple fibroids regarless of the surgical approach.

We recognise that the HA3D technique has limitations, a high number of specialised assistants are required for proper image sequencing. Furthermore, despite quick intraoperative use, the preoperative image processing requires several steps which inevitably take time. Also, it increases the cost of the procedure.

## Conclusion

In conclusion, incorporating HA3D imaging technology for the management of patients with gynaecologic surgical pathology is feasible. The adoption of HA3D imaging technology guide the surgeon to better plan the surgery. Difficult to locate uterine myomas may be missed during surgery, in these cases HA3D technology can be a valuable tool for the surgeon. Shorter operating times could result from the application of the HA3D technology. Further studies are needed to evaluate the clinical impact of this promising novel technology.

## Video scan (read QR)

https://vimeo.com/681242690/6dcc79ff02

**Figure:**